# Antiplatelet Aggregation Properties of Cirsilineol: A Novel Inhibitor of Blood Coagulation Factor Xa

**DOI:** 10.3390/ph16040588

**Published:** 2023-04-14

**Authors:** Go Oun Kim, Jong Beom Heo, Dong Ho Park, Gyu Yong Song, Jong-Sup Bae

**Affiliations:** 1 Research Institute of Pharmaceutical Sciences, College of Pharmacy, Kyungpook National University, Daegu 41566, Republic of Korea; rhdns9231@gmail.com; 2 College of Pharmacy, Chungnam National University, 99 Daehak-ro, Yuseong-gu, Daejon 34134, Republic of Korea; songmeom@gmail.com; 3 Department of Ophthalmology, School of Medicine, Kyungpook National University, Kyungpook National University Hospital, Daegu 41944, Republic of Korea; dongho_park@knu.ac.kr

**Keywords:** cirsilineol, FXa, platelet aggregation, thrombosis

## Abstract

A small natural substance called cirsilineol (CSL), which was discovered in the plant *Artemisia vestita*, is lethal to many cancer cells and has antioxidant, anticancer, and antibacterial properties. Here, we investigated the underlying mechanisms of the antithrombotic action of CSL. We demonstrated that CSL has antithrombotic efficacy comparable to rivaroxaban, a direct blood coagulation factor Xa (FXa) inhibitor employed as a positive control, in inhibiting the enzymatic activity of FXa and the platelet aggregation induced by adenosine diphosphate (ADP) and U46619, a thromboxane A2 analog. The expression of P-selectin, the phosphorylation of myristoylated alanine-rich C kinase substrate by U46619 or ADP, and the activation of PAC-1 in platelets were inhibited by CSL. Nitric oxide production was increased by CSL in ADP- or U46619-treated human umbilical vein endothelial cells (HUVECs), although excessive endothelin-1 secretion was suppressed. CSL demonstrated strong anticoagulant and antithrombotic effects in a mouse model of arterial and pulmonary thrombosis. Our findings suggest that CSL is a potential pharmacological candidate for a novel class of anti-FXa and antiplatelet medications.

## 1. Introduction

The activation of the intrinsic and extrinsic coagulation systems; adhesion, aggregation, and secretory functions of activated platelets; and thrombosis (one of the primary causes of death worldwide) are all directly associated with the activation of the coagulation systems [1]. Direct oral anticoagulants (DOACs) have emerged as a novel class of anticoagulants that have shown promising results in various thromboembolic disorders. Extensive preclinical and clinical trial data have demonstrated that DOACs offer several advantages over traditional anticoagulants, such as warfarin, including predictable pharmacokinetics, rapid onset of action, and fewer drug–drug interactions [2,3]. DOACs have been shown to be effective in preventing and treating venous thromboembolism, as well as reducing the risk of stroke and systemic embolism in patients with nonvalvular atrial fibrillation [4,5]. In addition, DOACs have been found to be safe and well-tolerated, with a lower incidence of major bleeding events compared to traditional anticoagulants. As a result, DOACs have been incorporated into international guidelines for the prevention and treatment of thromboembolic disorders [6,7,8]. Additionally, direct thrombin inhibitors and Factor Xa antagonists have become popular in several areas of cardiovascular medicine, replacing K-vitamin antagonists and, to some extent, low-molecular weight heparins. Unlike traditional anticoagulants, these drugs have different mechanisms of action and do not cause significant problems with paradoxical thrombosis. However, dabigatran has been linked to a higher incidence of myocardial infarction [9,10,11,12].

It is essential to conduct significant clinical trials on the correlation between anticoagulant and antiplatelet drugs. For example, the AGUSTUS study revealed that the addition of a Cox-1 inhibitor and P2Y12 inhibitor to the Xa antagonist offered an initial advantage but ultimately resulted in excessive bleeding [13], and it is generally accepted that an exhaustive evaluation of a patient’s risk and personalized treatment is essential to maximize the advantages of a possible combination of pharmaceutical methods [14].

Notably, several thromboembolic illnesses are also significantly impacted by platelet aggregation. Platelets are crucial for hemostasis and the beginning and continuing stages of thrombus development [15]. Inhibiting platelet activities has been demonstrated in large-scale randomized trials to enhance patient outcomes in various illness circumstances, including acute coronary syndromes, percutaneous revascularization treatments, and upper gastrointestinal bleeding. However, creating a new class of antiplatelet drugs is still necessary due to bleeding adverse effects and resistance issues [16,17]. 

**Figure 1:**
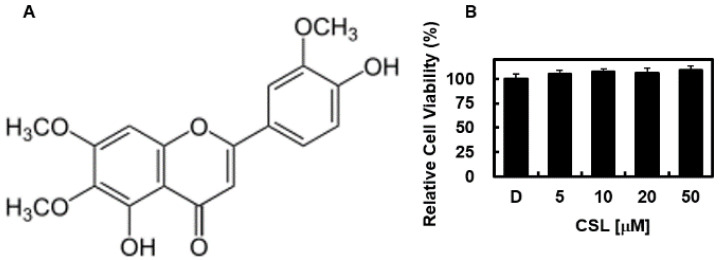
(**A**) Chemical structure of cirsilineol (CSL) and (**B**) the effect of CSL on cellular viability was measured using the MTT assay. The results represent the mean ± SD values from three independent experiments conducted in triplicate on different days.

Traditional Chinese herbal remedies have been used to treat several conditions, including diabetes, inflammatory disorders, liver disease, stroke, and cardiovascular disease [18]. However, scientific research does not support these advantages [18,19]. Research on conventional herbal and botanical medicines is expanding quickly, and natural products are becoming increasingly popular due to their low cost and lack of adverse effects [18]. The flavone bioactive substance cirsilineol (CSL, Figure 1), 4,5-dihydroxy-3,6,7-trimethoxyflavone, is found in the Chinese and Tibetan plant *Artemisia vestita* (Asteraceae). CSL is a powerful anti-inflammatory, hypnotic, anticancer, antibacterial, and anti-anxiety medication that demonstrates cytotoxicity against several cancer cells [20,21,22,23]. However, FXa- and platelet-related CSL antithrombotic actions have not yet been documented. This study aimed to determine the antithrombotic properties of CSL by examining FXa activity, blood clotting time, and platelet function. We further characterized the antithrombotic properties of CSL in mouse models. We observed, for the first time, that CSL has antithrombotic effects by downregulating FXa and platelet activities.

## 2. Results

### 2.1. Effects of CSL on Cellular Viability, Clotting Time In Vitro and Ex Vivo

**Figure 2:**
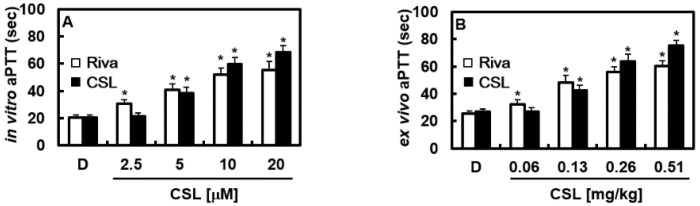
Effects of CSL on clotting time. (**A**) Platelet-poor plasma (PPP) was incubated with the indicated concentrations of CSL or rivaroxaban (Riva) for 15 min. (**B**) One hour after administering DMSO, CSL, or Riva, blood was collected from the mice, and PPP was obtained. Then, in vitro aPTT (**A**) or ex vivo aPTT was measured, as described in the Methods sections. Data are shown as mean values ± SD of five independent experiments. * *p* < 0.05 vs. D. D = 0.2% DMSO, used as the vehicle control.

First, the potential cytotoxicity of CNS on HUVECs was assessed using the 3-(4,5-dimethylthiazol-2-yl)-2,5-diphenyltetrazolium bromide (MTT) assay. As shown in Figure 1B, CSL-treated cells with different concentrations (5–50 μM) had no change in cell viability. The activated partial thromboplastin time (aPTT) and prothrombin time (PT), two coagulation parameters altered by CSL in vitro, were assessed first. We used the direct FXa inhibitor rivaroxaban as a positive control for our experiments. CSL considerably slowed the aPTT at doses between 5 and 20 M (Figure 2A). In the aPTT experiments, rivaroxaban at 8.38 μM and CSL at 7.23 μM, respectively, doubled the clotting time. Each mouse group (*n* = 5) received intravenous injections of CSL for 4 consecutive days to validate the in vitro results in an ex vivo experiment. Our findings demonstrated that CSL dramatically increased blood clotting time in a concentration-dependent manner (Figure 2B). In the ex vivo aPTT assays, treatment with CSL at 0.25 mg/kg and rivaroxaban at 0.29 mg/kg, respectively, doubled the clotting time. The average blood volume was determined to be 2 mL based on an estimated circulating blood volume for mice of 72 mL/kg [24] and an average weight of 27 g for each animal used in this investigation. As a result, the estimated concentration of CSL in the peripheral blood after each injection (0.06, 0.13, 0.26, or 0.51 g/kg) was 2.5, 5, 10, or 20 μM, respectively. Notably, we discovered that when CSL or rivaroxaban was administered to mice, the PT did not change (data not shown).

### 2.2. Effects of CSL on Platelet Aggregation In Vitro

**Figure 3:**
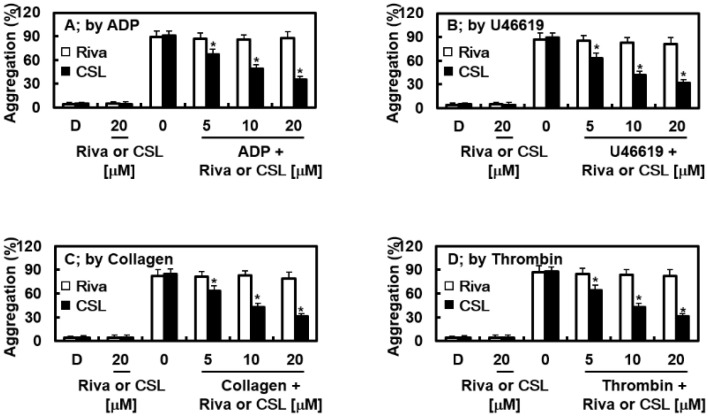
Effects of CSL on agonist-induced platelet aggregation. Platelet-rich plasma (PRP) was preincubated for 5 min with different concentrations of CSL, Riva, or the vehicle. Platelet aggregation was initiated with ADP ((**A**) 10 μM), U46619 ((**B**) 6 μM), collagen ((**C**) 1 µg/mL), or thrombin ((**D**) 3 U/mL), and platelet aggregation was recorded using an aggregometer. Data are shown as mean values ± SD of five independent experiments. * *p* < 0.05 vs. D. D = 0.2% DMSO, used as the vehicle control.

Human platelet-rich plasma (PRP) was used to study agonist-induced platelet aggregation to ascertain whether CSL slows this process. Rivaroxaban did not affect the degree of platelet aggregation induced by the different agonists, while CSL significantly suppressed the platelet aggregation induced by ADP (10 μM), collagen (1 μg/mL), thrombin (3 U/L), and U46619 (6 μM) in a concentration-dependent manner (Figure 3).

### 2.3. Effects of CSL on Animal Models of Arterial and Pulmonary Thrombosis 

**Figure 4:**
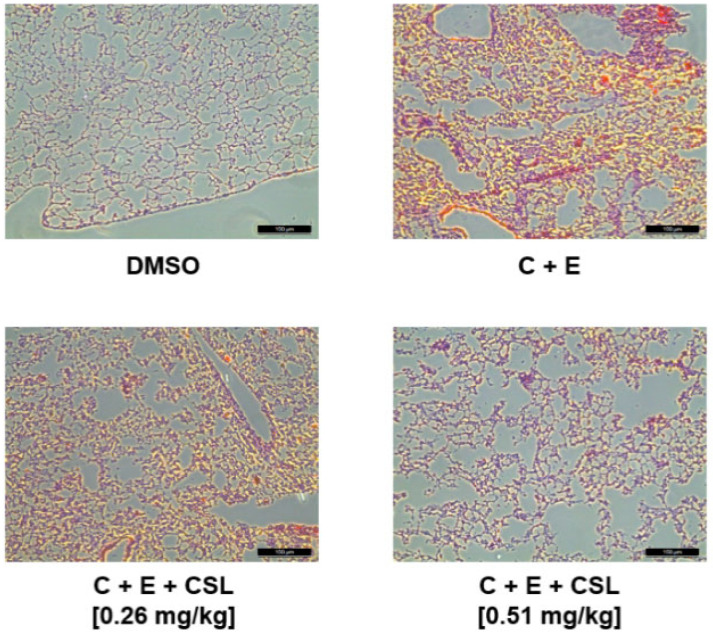
Effects of CSL on microvascular thrombosis. Representative H&E sections of lungs from mice treated as labeled. C + E: Treatment of collagen with epinephrine. Magnification: ×200. Scale bar: 100 μm.

We used an animal model of FeCl_3_-induced carotid artery thrombosis [25] to examine the CSL-mediated antithrombotic effects in vivo (Iuhara et al., 2008). The impact of CSL on the timing and magnitude of FeCl_3_-induced thrombus development is shown in Table 1. Large thrombi developed more quickly due to FeCl_3_-induced endothelial damage. However, CSL slowed this proliferation significantly. Additionally, the in vivo pulmonary thrombosis model demonstrated extremely significant pulmonary thrombosis with immediate paralysis after receiving an injection of a collagen and epinephrine mixture. CSL significantly reduced mortality and the formation of scored thrombus in mouse lung tissues compared to treatment with a collagen and epinephrine mixture alone (Figure 4, Table 1).

### 2.4. Effects of CSL on the Catalytic Activity and Production of FXa In Vitro

**Figure 5:**
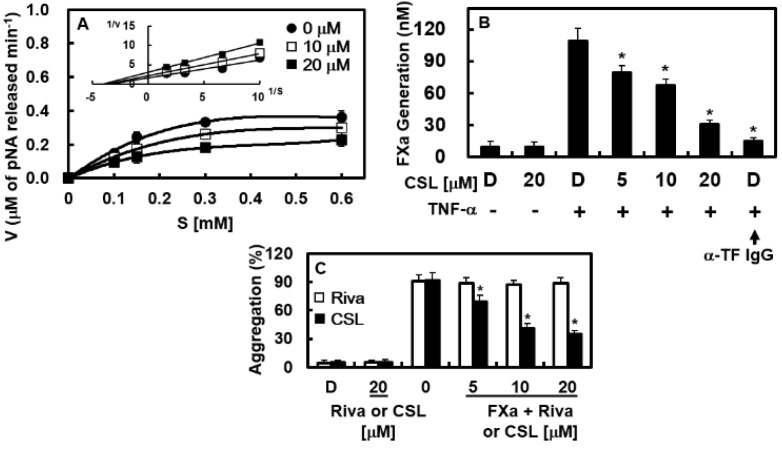
Effects of CSL on the catalytic activity and production of FXa. (**A**) Lineweaver–Burk plot kinetics (inset) and Michaelis–Menten plot demonstrate a mixed inhibitory pattern for CSL. Plots show the average values of five independent measurements. (**B**) HUVECs pre-incubated with the indicated concentrations of CSL for 10 min were stimulated with TNF-α (10 ng/mL) for 6 h and incubated with FX (175 nM) and FVIIa (10 nM) with/without anti-tissue factor antibody (25 µg/mL). The production of FXa was characterized as described in the Methods section. (**C**) PRP was preincubated for 5 min with different concentrations of CSL, Riva, or the vehicle. Platelet aggregation was initiated with FXa (52 µg/mL), and the platelet aggregation was recorded using an aggregometer. Data are shown as mean values ± SD of five independent experiments. * *p* < 0.05 vs. TNF-α alone (**B**) or FXa (**C**). D = 0.2% DMSO, used as the vehicle control.

The effects of CSL on the activity and generation of FXa were identified to examine the mechanisms of CSL-mediated coagulation and platelet aggregation regulation. On substrate S-2222, CSL inhibited the catalytic activity of FXa in a concentration-dependent manner. The Michaelis–Menten plots for CSL revealed slopes of 0.568 and 0.759 for CSL at 10 and 20 μM, respectively. A slope of 0.470 was revealed for the control without CSL (Figure 5A). The inhibition of FXa by CSL is depicted in the Lineweaver–Burk plots in Figure 5A (inset), which shows a mixed-type inhibitory pattern. CSL induced a decrease in the V_max_, with a concomitant increase in the K_m_ value of FXa on S-2222. We discovered that CSL inhibited FXa activity toward S-2222 at a K_i_ value of 3.71 μM. (Table 2). A comparison of the selectivity of CSL for FXa with other representative human enzymes in the coagulation cascade is also shown in Table 2. CSL was highly selective for FXa, with a selectivity ratio (based on their respective K_i_ values) of >300 for FXa compared to other enzymes examined. Because TF expression was required for the FVIIa-mediated activation of FX in TNF-α-stimulated human umbilical vein endothelial cells (HUVECs) [26,27], we also studied the effects of CSL on this process. The activation rate of FX by FVIIa was 11.4 times higher when TNF-α stimulation was applied to HUVECs to promote TF expression (109.1 ± 5.1 nM vs. 9.6 ± 0.8 nM) (Figure 5B). FX activation was notably impeded by the anti-TF IgG treatment (15.2 ± 2.9 nM, Figure 5B). Our findings showed that pre-incubation with CSL inhibited FX activation in a concentration-dependent manner (Figure 5B). Petzold et al. recently demonstrated that FXa could cause platelet aggregation [28]. As a result, we identified the contribution of CSL to FXa-mediated platelet aggregation. In Figure 5C, it is demonstrated that CSL dramatically reduced platelet aggregation brought on by FXa.

### 2.5. Effects of CSL on the Activation of Protein Kinase C and Mobilization of Intracellular Calcium

**Figure 6:**
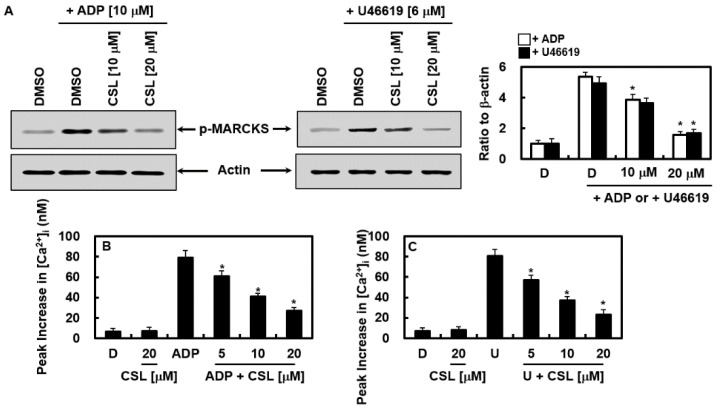
Effects of CSL on activation of protein kinase CPKC activation and mobilization of intracellular calcium. (**A**) Rinsed platelets were incubated with DMSO or CSL (10 or 20 μM) for 10 min at 37 °C, and stimulation of ADP (10 μM, left) or U46619 (6 μM, right) was given for 1 min. The phosphorylation level of MARCKS in the platelets was determined with western blotting. Representative images from each group are shown (*n* = 3). Densitometric intensities of each signaling pathway component normalized to β-actin or total protein. (**B**,**C**) Human platelets preloaded with Fura-2 were incubated with DMSO (D) or CSL with extracellular Ca^2+^ (1 mM) for 10 min at 37 °C, and ADP ((**B**) 10 μM) or U46619 ((**C**) 6 μM) stimulation was given to induce an increase in [Ca^2+^]_i_. Data are shown as mean values ± SD of five independent experiments. * *p* < 0.05 vs. ADP (**A**,**B**) or U46619 (**A**,**C**) alone. D = 0.2% DMSO, used as the vehicle control.

Next, the molecular mechanism by which CSL controlled platelet aggregation was identified. We examined the effects of CSL on PKC activation by examining the phosphorylation level of myristoylated alanine-rich C kinase substrate (MARCKS), a significant phosphorylation substrate of PKC in human platelets. Both protein kinase C (PKC) activation and an increase in cytosolic Ca^2+^ can cause platelet aggregation [29]. Indeed, CSL treatment prevented MARCKS from becoming phosphorylated in response to ADP and U46619, showing that CSL inhibited PKC (Figure 6A). Phospholipase C hydrolyzed phosphatidylinositol 4,5-bisphosphate into inositol-1,4,5-triphosphate and diacylglycerol when the platelets were activated by agonists, which leads to an increase in cytosolic Ca^2+^ and the activation of PKC, respectively [30]. Ca^2+^ and PKC worked together to secrete granules and activated the glycoprotein PAC-1 (GPIIb/IIIa), the ultimate receptor for platelet aggregation [30]. ADP- and U46619-induced elevations in [Ca^2+^]_i_ were inhibited by CSL administration (Figure 6B,C). Our findings showed that CSL suppressed [Ca^2+^]_i_ and PKC activation, reducing platelet aggregation.

### 2.6. Effects of CSL on the Expression of P-Selectin and PAC-1

**Figure 7:**
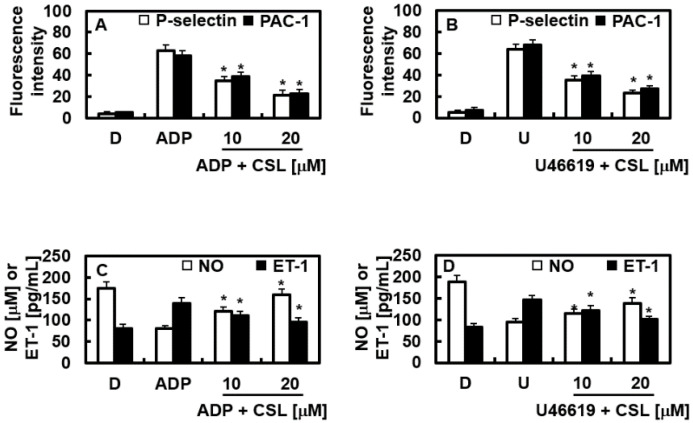
Effects of CSL on the expression of P-selectin and PAC-1 and the production of NO and ET-1. (**A**) Rinsed platelets were incubated with DMSO or CSL (10 or 20 μM) for 3 min at 37 °C, and stimulation of ADP ((**A**,**C**) 10 μM) or U46619 ((**B**,**D**) 6 μM) was given for 6 min. The expressions of P-selectin and PAC-1 (**A**,**B**) and the production of NO and ET-1 (**C**,**D**) were measured as described in the Methods section. Data are shown as mean values ± SD of five independent experiments. * *p* < 0.05 vs. (**A**,**C**) ADP or (**B**,**D**) U46619 alone. D = 0.2% DMSO, used as the vehicle control.

P-selectin is translocated to the exterior membrane when platelets are activated, and fibrinogen connects PAC-1 between adjacent platelets, causing aggregation [31]. Figure 7A,B show that CSL reduced the expression of P-selectin and PAC-1 in the platelets activated by ADP or U46619. CSL inhibited platelet aggregation by lowering the platelet expressions of P-selectin and PAC-1.

### 2.7. Effects of CSL on NO and ET-1

An important endogenous vasodilator, NO prevents platelet aggregation and protects the arterial wall. A potent vasoconstrictor called ET-1 is produced from the vascular endothelium and secretes NO [32,33,34]. The effects of CSL on the levels of NO and ET-1 were found to provide a further mechanistic understanding of the CSL-mediated inhibition of platelet aggregation. It was noted that adequate regulation of vasomotion from the controlled production of NO and ET-1 enabled adequate maintenance of vascular homeostasis [35,36]. The results demonstrated that CSL returned the increased production of ET-1 and returned the decreased NO secretion caused by ADP or U46619 to their baseline levels (Figure 7C,D).

## 3. Discussion

Several medications that reduce platelet aggregation are now widely used to treat thrombotic diseases alone or in conjunction with other medicines that inhibit platelets. However, their long-term usage is regularly known to have adverse effects [37,38]. Our discovery that CSL efficiently reduced ADP-, U46619-, collagen-, or thrombin-induced platelet aggregation points to a unique possibility for CSL in creating clinically useful medications with fewer side effects. FXa exhibits an essential function in the coagulation cascade [39]. We hypothesized that the selective inhibition of FXa might help as a potentially effective method for new antithrombotic drugs, especially in light of the position of FXa at the converging step between the extrinsic and intrinsic coagulation pathways. We noted that the inhibitors directly targeting thrombin demonstrated a narrow therapeutic index [40,41]. Additionally, it was anticipated that CSL (with a molecular weight of 344.32), which is smaller than other FXa inhibitors, would have several advantages over increased anticoagulant molecules, including low production costs and low immunogenicity. 

Platelet aggregation and clotting time are the most often utilized criteria for assessing the effectiveness of novel antithrombotic medications [42]. In our studies, aPTT and PT tests were used to assess the effects of CSL on the intrinsic and extrinsic coagulation pathways, respectively, to validate its anticoagulant effects. The aPTTs in mice were prolonged by CSL longer than by the vehicle treatment, and the CSL treatment did not appear to promote PT. It is unknown why CSL affected the aPTT but not the PT. It was thought that the antithrombotic dosages of CSL caused more hemostasis disruption or that CSL inhibited the intrinsic coagulation pathway-specific factors (e.g., factor XIa, XIIa, or VIIIa as a cofactor for FXa), but not factor VIIa, which was specific to the extrinsic coagulation pathway. The ex vivo aPTT was likewise lengthened by CSL administration in a dose-dependent manner. Inhibiting the platelet aggregation brought on by ADP, collagen, U46619, or thrombin therapy demonstrates that CSL also acts as a selective platelet aggregation inhibitor. 

We demonstrated that CSL had antithrombotic properties comparable to rivaroxaban, including the ability to decrease FXa synthesis and activity, lengthen blood clotting time, and delay thrombogenesis and thrombogenic time. The platelet aggregation caused by ADP or U46619 was not inhibited by rivaroxaban, but CSL suppressed it in a concentration-dependent manner. Our mechanistic research showed that their antiplatelet activities were mediated by PKC activation, intracellular Ca^2+^ mobilization, and P-selectin and PAC-1 expression levels. Therefore, CSL has antiplatelet potential by inhibiting platelet aggregation, coagulation times, cytosolic Ca^2+^ mobilization, FXa activation and generation, the phosphorylation of MARCKS through the PKC pathway, and the expression of P-selectin and PAC-1. Additionally, CSL decreased the synthesis of ET-1, a vasoconstrictor, and improved the secretion of the NO vasodilator. The activation, adhesion, and aggregation processes of platelets are involved in the mechanism that causes the coagulation event [39,43]. Coagulation is frequently initiated by an injury that damages the endothelium lining. Blood exposure to the subendothelial space of blood vessels triggers changes in the platelets and subendothelial TF to FVII exposure, ultimately leading to fibrin formation [39,43]. As the thrombus grows, the active intrinsic route, which comprises FVIII, FIX, and the hemophilia factors, attracts more platelets and amplifies the coagulation cascade [39]. A crucial stage in amplifying the coagulation cascade is to provide a thrombogenic surface provided by the platelets and endothelial cells. Therefore, for the most crucial roles of antithrombotic and antiplatelet actions, the activation and altered behavior of the platelets are the molecular targets of both the blood coagulation and the platelet aggregation pathways. 

Petzold et al. recently demonstrated that rivaroxaban decreased platelet aggregation using blood taken from patients on rivaroxaban treatment [28]. According to previous studies, rivaroxaban has consistently been shown not to affect platelet aggregation brought on by collagen, ADP, thromboxane A2, or thrombin [27,44,45,46,47]. The current study confirmed this. The origin of the blood sample is one theory as to why there is a difference. Blood samples included in the study by Petzold had clinical signs of thrombosis, hypertension, and diabetes, all of which are cardiovascular illnesses. Once rivaroxaban is absorbed into the vascular bloodstream, it may interact with other drugs, changing its original qualities. Modified rivaroxaban may impact platelet aggregation by triggering or squelching unidentified platelet aggregation signaling pathways. According to Petzold et al., FXa is a potent platelet agonist that causes platelet aggregation. The results of this investigation demonstrated that CSL could inhibit FXa-mediated platelet aggregation, indicating that CSL acts as an antiplatelet agent via an FXa-mediated signaling cascade. 

Once rivaroxaban is absorbed into the vascular blood system, it could interact with other medications, alternating its original properties. Altered rivaroxaban might affect platelet aggregation by activating or inhibiting unknown platelet aggregation signaling pathways. Petzold et al. also identified FXa as a potent platelet agonist, resulting in platelet aggregation. This study showed that CSL could suppress FXa-mediated platelet aggregation, indicating that CSL exerts an antiplatelet effect through the FXa-mediated signaling cascade. 

In conclusion, we showed that CSL is a novel inhibitor of intrinsic blood coagulation pathways and an anti-FXa and antiplatelet molecule. Our findings might aid in the development of novel therapeutic approaches and avoiding coagulation-related thrombotic diseases.

## 4. Materials and Methods

### 4.1. Cell Culture and Reagents 

HUVECs were purchased from Cambrex Bio Science (Charles City, IA, USA) and maintained as previously described [48,49]. Abnova (Taipei, Taiwan) and Bayer HealthCare (Leverkusen, Germany) provided tumor necrosis factor (TNF) and rivaroxaban, respectively. Collagen and CSL were purchased from Sigma-Aldrich (St. Louis, MO, USA). The Calbiochem-Novabiochem Corp. provided U46619 (San Diego, CA, USA). The following substances were purchased from Haematologic Technologies: plasmin, FVIIa, FX, FXa, activated protein C, tPA, trypsin, and thrombin (Essex Junction, VT, USA). Fisher Diagnostics provided reagents for thromboplastin aPTT and PT assays (Middletown, VA, USA). The chromogenic substrates were all purchased from Chromogenix AB (Mölndal, Sweden) and were designated S-2222 for trypsin, S-2228 for tPA, S2238 for thrombin, S-2251 for plasmin, S-2366 for activated protein C, and S-2765 for FXa. BD Pharmingen (BD Biosciences, San Diego, CA, USA) provided the anti-CD61-FITC, anti-CD62PPE, anti-PAC-1, and anti-CD61-PE antibodies. Santa Cruz Biotechnology provided anti-tissue factor (TF) antibodies (Santa Cruz, CA, USA).

### 4.2. Animal Care and Blood Correction

Male C57BL/6 mice aged 7 weeks (mean weight: 27 g) were purchased from Orient Bio Co., in Sungnam, Republic of Korea, and were subjected to a 12-day acclimatization phase [48,49]. The Guidelines for the Care and Use of Laboratory Animals, approved by Kyungpook National University, were followed when handling the mice for the investigations (Institutional Review Board (IRB) No. KNU 2021-107). Compounds were administrated intravenously at 200 μL (10% *v*/*v*) and the plasma from mice was collected, 800 μL of whole blood from the mouse heart, centrifuged at 1500× *g* for 15 min and the plasma stored at −80 °C. Proteins in plasma were stable when stored at −80 degrees, and experiments proceeded immediately after plasma isolation.

### 4.3. Care Preparation of Human Plasma and Platelets

Ten fasting healthy volunteers (aged 24–28; six women and four men) who were free of allergies, cardiovascular problems, carbohydrate, or lipid metabolic abnormalities and had not taken any morning medication had their blood samples taken. None of our subjects used addictive drugs or antioxidant food supplements, and their diets were well balanced. The plasma from the blood samples was immediately spun down (1300× *g* for 15 min) to separate it from sodium citrate (10.9 mM) and pooled for the following investigations. Next, human blood samples were spun down at room temperature to rapidly separate the PRP. The PRP concentration was adjusted to 1 × 10^9^ platelets/mL using a hemocytometer. After being rinsed once in HEPES buffer containing 1 mM CaCl_2_, the PRP pellet was maintained at room temperature for an additional 30 min. Then, 10 mL of the platelet samples were utilized to measure each clotting time point. The IRB of Kyungpook National University Hospital (Daegu, Republic of Korea) approved this study protocol (KNUH 2012-01-010).

### 4.4. In Vitro Coagulation Assay

Plasma was taken by centrifuging blood (2000× *g* for 10 min) at room temperature following dimethyl sulfoxide (DMSO) or CSL administration for 1 h to assess PT and aPTT using a thrombotimer (Behnk Elektronik, Norderstedt, Germany) [50]. Micronized silica was utilized as an activator for the aPTT test. Briefly, 100 μL of platelet-poor plasma (PPP) and 100 μL of aPTT assay reagent were combined for 1 min at 37 °C, 100 L of CaCl_2_ (20 mM) was added, and the clotting times were noted. A total of 200 μL of the PT assay reagent was incubated for 10 min at 37 °C to conduct the PT assays and mixed with 100 μL of PPP for 3 min at 37 °C. Next, the clotting time was measured.

### 4.5. In Vitro Platelet Aggregation Assay 

CSL dissolved in DMSO was incubated with human PRP for a specified time (1, 3, 5, or 10 min). Next, PRP was activated by the addition of collagen (1 μg/mL), ADP (10 μM), U46619 (6 μM), or thrombin (3 U/mL), and the amount of platelet aggregation was measured using an aggregometer (Chronolog, Havertown, PA, USA).

### 4.6. Inhibition of FXa Amidolytic Activity 

CSL was dissolved in 50 mM Tris buffer, which has a pH of 7.4, and was added to 150 μL of FXa solution (1 U/mL). This mixture was incubated for 1 min at 37 °C. Additionally, 150 μL of S-2222 solution (1.5 mM) was blended, and for 20 min, the changes in absorbance were observed spectrophotometrically at 405 nm. We used the slope of the curve (V_i_) and the absorbance–time curve to determine enzyme activity. Equation (1) was used to compute the CSL inhibitory rates:

Inhibitory rate (%) = (V_0_ − V_i_)/V_0_ × 100 (1)

where V_i_ and V_0_ represent the slopes of the samples and the vehicle, respectively.

### 4.7. Inhibitory Constant for FXa 

K_i_ values were calculated based on chromogenic substrate experiments. CSL was employed in each assay at a minimum of seven concentrations in duplicate to obtain an inhibition curve. A total of 50 μL of substrate was combined with 25 μL of each chemical solution, inhibitor solution, or blank buffer. Before the plate was allocated into the Labsystems IEMS microtiter plate reader (Cergy Pontoise, France), 25 μL of the enzyme solution was mixed. The plate was allowed for 1 h at 37 °C before analysis. We continuously observed the formation of *p*-nitroaniline produced when the substrate was hydrolyzed using spectrophotometry at 405 nm. The maximum initial reaction rates were computed as the millioptical density per minute. A Dixon plot of 1/V_max_ versus inhibitor concentration was used to fit the curve and determine the K_i_ values.

### 4.8. Production of FXa on the Surface of HUVECs 

Confluent HUVEC monolayers developed on 96-well culture plates that were stimulated with TNF-α (10 ng/mL) for 6 h after exposure to the relevant CSL concentration for 10 min. The cells were exposed to FVIIa (10 nM) in buffer A (10 mM HEPES buffer, pH 7.45; 11 mM glucose, 150 mM NaCl, and 4 mM KCl), supplemented with 5 mM CaCl2, and 5 mg/mL BSA, for 5 min at 37 °C with/without anti-TF IgG (25 μg/mL). Following the addition of FX, the mixture was given a final reaction mixture volume of 100 μL before being incubated for 15 min. The reaction was stopped by adding buffer A supplemented with 10 mM EDTA, and the amount of FXa produced was determined by adding S-2765. Spectrophotometric monitoring of the absorbance over a 2 min period was performed at 405 nm. Initial rates of color development were converted into FXa concentrations using a standard curve built with known concentrations of pure human FXa.

### 4.9. Cell Viability Assay 

A 3-(4,5-dimethylthiazol-2-yl)-2,5-diphenyltetrazoliumbromide (MTT) assay was used to measure cell viability [48,49]. For cell cultivation, 96-well plates were used at a density of 5 × 10^3^ cells per well. After a day, the cells were washed with fresh media and treated with CSL. The cells were rinsed, 100 μL of MTT (1 mg/mL) was added, and they were cultured for another 48 h before being tested. Finally, 150 μL of DMSO was added to the reaction to solubilize the formazan salt that had been created. The amount of formazan salt formed was measured spectrophotometrically at 540 nm.

### 4.10. Western Blotting

SDS-PAGE was used to isolate identical amounts of protein from the HUVEC extracts, and the protein was then electrotransferred onto an immobilon membrane (Millipore, Billerica, MA, USA). The membranes underwent blocking, anti-phospho-MARCKS (Santa Cruz, CA, USA) incubation for 1.5 h at room temperature, HRP-conjugated secondary antibody incubation, and ECL detection. We employed anti-(-Actin (1:1000, Santa Cruz, CA, USA) as a loading control. ImageJ Gel Analysis tool (NIH, Bethesda, MD, USA) was used for concentration analyses.

### 4.11. Measurement of Intracellular Ca^2+^ Mobilization

The amount of intracellular Ca^2+^ ([Ca^2+^]_i_) of platelets was measured as previously described [51]. A final concentration of 5 × 10^7^ platelets/mL in Tyrode’s solution without Ca^2+^ was achieved after platelets were loaded with Fura 2-AM (3 μM) at 37 °C for 30 min, washed twice, and suspended. The suspension of platelets received a calcium (1 mM) addition before stimulation with platelet activators for 1 min. Grynkiewicz et al. [52] proposed an equation for calculating the [Ca^2+^]_i_ by measuring the intensity of fluorescence signals (excitation/emission wavelengths: 339/500 nm) obtained from the platelets.

### 4.12. Measurement of PAC-1 and P-Selectin Expression

PAC-1 and P-selectin expression levels were assessed by modifying accepted techniques [53]. Briefly, 20 μL of DMSO or CSL (10 to 20 μM) were incubated with 0.5 mL of cleaned platelets (2 × 10^8^ platelets/mL) for 3 min. The platelet suspension was refrigerated at 4 °C after stimulation with ADP (10 μM) or U46619 (6 μM) for 6 min at 37 °C. Subsequently, 50 μL of the sample was mixed with activation-specific anti-PAC-1 and anti-CD61-PE in saturated concentrations, and the mixture was then incubated for 25 min in the dark to determine the expression of PAC-1. Anti-CD62P-PE and anti-CD61-FITC were combined with 50 μL of the sample and incubated for 25 min in the dark to determine the expression of P-selectin. Samples that had been quench-diluted (<4-fold) were examined using a flow cytometer (BD, Biosciences, San Diego, CA, USA). Populations of platelets were gated on cell size with forward scatter vs. side scatter and CD61 positivity to distinguish platelet populations from background noise. Finally, 5000 events were evaluated for each sample. The mean fluorescence intensities from each channel were expressed using BD Accuri C6 software (BD Biosciences, San Diego, CA, USA). Five distinct samples of donors were used for the measurements.

### 4.13. Quantification of Nitrogen Monoxide (NO) and Endothelin-1 (ET-1)

A commercially available ELISA kit was used to measure the generation of NO and ET-1 (R&D Systems, Minneapolis, MN, USA) in the cell culture medium.

### 4.14. Arterial Thrombosis Animal Model 

A mouse model of FeCl_3_-induced thrombosis was created and used. Male C57BL/6 mice were starved for the entire night before receiving intravenous injections of the relevant drug in 0.2 mL of DMSO and were allowed to sleep. The carotid artery of the animal was exposed. The adventitial surface was covered with a cotton thread (200 μm in diameter) soaked in FeCl_3_ solution (0.25 M) for 5 min. The wound was flushed with saline solution once the thread had been removed. The development of thrombus was observed at 35 °C using 3D imaging. We measured the size and rate of thrombus formation, and we graded our results as follows: no thrombus or small thrombus (75–50 μm), medium thrombus (150–100 μm), massive thrombus (300–200 μm), and more than three thrombi. It was also noted how long it took for an extensive thrombus to obstruct the carotid artery following endothelial damage caused by FeCl_3_.

### 4.15. Acute Pulmonary Thrombosis Induced by Combined Treatment of Collagen and Epinephrine in an Animal Model

Mice were separated into several groups after being fasted overnight (10 animals each). CSL was dissolved in DMSO and given intravenously to each mouse. After 1 h, the animals received an injection of collagen and epinephrine mixture (500 μg/kg each) to induce acute thrombosis [54,55]. Each mouse was closely observed for 15 min to see if it had recovered from the acute thrombosis challenge, had been paralyzed, or had passed away. H&E-stained lung tissue slices from five mice were randomly selected for the histologic investigation to determine the extent of pulmonary thrombus development. The number of arteries greater than 20 μm in diameter with any detectable thrombus was counted in each of the five view fields randomly selected from the left lobe in each section (one section per mouse). Twenty-five fields for each group were compared as a result. A total of 60–80 vessels in each segment were imaged using the system (Leica, Germany). The results were graded as the number of thrombi per 25 mm^2^ lung field as previously described [56].

### 4.16. Statistical Analysis

The data are presented as the mean value ± standard deviation (SD) of five separate experiments conducted in duplicate. Tukey’s tests were run as a post-hoc examination of the differences between the various groups after one-way analysis of variance revealed a significant difference between the groups. A *p*-value of <0.05 was considered statistically significant.

## Figures and Tables

**Table 1:** Effects of CSL on the model of arterial and pulmonary thrombosisa.

**Time to Large Thrombus Formation (min)**				
**mg/kg**	**Rivaroxaban**		**CSL**	
DMSO	7.4 ± 0.5		7.1 ± 0.4	
0.13	15.3 ± 1.1 *		13.1 ± 1.1 *	
0.26	21.8 ± 1.9 *		27.2 ± 2.1 *	
0.51	44.3 ± 3.1 *		51.4 ± 3.1 *	
**Scored thrombus formation**				
**mg/kg**	**Rivaroxaban**		**CSL**	
DMSO	3.9 ± 0.1		4.1 ± 0.1	
0.13	3.5 ± 0.1 *		3.7 ± 0.2 *	
0.26	3.1 ± 0.3 *		2.7 ± 0.1 *	
0.51	2.3 ± 0.1 *		1.8 ± 0.1 *	
**In vivo pulmonary thrombosis model** **(Mortality % and Scored thrombus formation, *n* = 20)**				
**mg/kg**	**Rivaroxaban**		**CSL**	
	%	Thrombi	%	Thrombi
DMSO	0	0	0	0
C + E	95	3.9 ± 0.3	95	4.1± 0.2
0.13	85 ^#^	3.1 ± 0.1 ^#^	85 ^#^	3.5 ± 0.1 ^#^
0.26	75 ^#^	2.6 ± 0.1 ^#^	80 ^#^	2.5 ± 0.1 ^#^
0.51	55 ^#^	1.9 ± 0.2 ^#^	60 ^#^	1.8 ± 0.2 ^#^

All inhibitors were dissolved in 0.2% dimethyl sulfoxide (DMSO). Values represent the means ± SD (*n* = 5). * *p* < 0.05 compared to DMSO or ^#^ *p* < 0.05 compared to C + E (collagen and epinephrine).

**Table 2:** Enzyme kinetics and selectivity of CSL against different human enzymes.

	MA	
Enzyme	K_i_^a^	Ratio ^b^
Factor Xa	3.70 ± 0.19	1
α-Thrombin	>300	>100
Trypsin	>300	>100
Plasmin	>300	>100
Protein Ca	>300	>100
tPA ^c^	>300	>100

^a^ K_i_ is represented by the mean ± SD (*n* = 5), μM. ^b^ Ratio = K_i_ enzyme/K_i_ Factor Xa. ^c^ tPA, tissue plasminogen activator.

## Data Availability

The data presented in this study are available upon reasonable request from the corresponding author.
